# Establishing clinical interpretability of automatic speech assessments in Alzheimer's disease

**DOI:** 10.1002/alz70856_097515

**Published:** 2025-12-24

**Authors:** Jelena Curcic, Iris Nowenstein, Bryndís Bergþórsdóttir, Gunnar Thor Örnólfsson, Hinrik Hafsteinsson, Jessica Robin, Daniel Garellick, Jennifer Sorinas, Kristin Hannesdottir

**Affiliations:** ^1^ Novartis Biomedical Research, Basel, Switzerland; ^2^ University of Iceland, Reykjavík, Iceland; ^3^ Reykjavík University, Reykjavík, Iceland; ^4^ Cambridge Cognition, Cambridge, United Kingdom; ^5^ Novartis Biomedical Research, Cambridge, MA, USA

## Abstract

**Background:**

Following advances in language technology over the last decade, a rapidly growing body of research shows promising results on the potential of automatic speech and language analysis in the early detection and monitoring of Alzheimer's disease. To critically assess the outputs of automatic analyses, it is necessary to investigate the clinical interpretability of automatically extracted speech and language features and relate them to patient‐centered outcomes such as speech fluency and word‐finding difficulties. Additionally, a key component of generalizability and equitable access to language‐based health technology is to expand the research beyond high‐resource languages such as English.

**Method:**

48 native Icelandic speakers participated in a non‐interventional, cross‐sectional study and were grouped into four cohorts: cognitively healthy controls (amyloid negative), cognitively healthy (amyloid positive) pre‐symptomatic, mild cognitive impairment and mild Alzheimer's disease [Curcic et al., JMIR, 2022]. All participants underwent six different oral picture description tasks. The picture description was recorded for each individual participant at three sessions: one baseline (morning), two follow‐up 4 to 32 days later (morning and evening). Both acoustic and lexical features were extracted from audio and text (transcripts) automatically using a speech activity detector and language‐specific natural language processing tools for part‐of‐speech tagging. Two speech‐language pathologists (SLPs) conducted expert ratings of each picture description recording along various speech and language dimensions (e.g. fluency, motor speech characteristics and content depth).

**Result:**

Recordings from this cross‐sectional study are currently being analyzed. Agreements between two SLPs within each rating dimension will be calculated. Correlations between automatically extracted speech and language features and average of two expert SLP‐based ratings will be presented and the cohort classification outputs of both methods compared.

**Conclusion:**

Many conventional clinical trial endpoints in Alzheimer's disease are subjective, lack sensitivity and are burdensome to administer. The promise of automatic language and speech analytics has been touted as providing brief, objective, and non‐invasive assessments targeting core clinical features of Alzheimer's disease. This study marks an important step in turning such promise into reality and helping advance this game‐changing technology into global clinical trials.

